# A patient with *DAX1*  mutation presenting with elevated testosterone in early infancy

**DOI:** 10.1007/s12519-019-00236-4

**Published:** 2019-03-20

**Authors:** Juan Ge, Tang Li

**Affiliations:** 1 Medical Department, Qingdao University, Qingdao, China; 2 Department of Pediatric Endocrinology and Genetic Metabolic Diseases, Qingdao Women and Children’s Hospital, Qingdao, China

Dosage-sensitive sex reversal-adrenal hypoplasia congenita critical region on the X chromosome 1 (DAX1) deficiency is a rare disorder presents X-Linked adrenal hypoplasia congenital, impaired sexual development and infertility. Mutational gene of DAX1 was identified as *NROB1* [1] which expresses in adrenal gland and hypothalamic–pituitary–gonad axis, restrains progenitor stem cells from differentiating into steroidogenic cells prematurely. Therefore, DAX1 deficiency usually manifests primary adrenal insufficiency and hypogonadotropic hypogonadism. However, we observed a patient whose testosterone elevated in early infancy.

With uneventful pregnancy and birth history, the patient got intractable jaundice. There is a maternal uncle died young suddenly in the family history. Biochemical findings showed persistent hyponatremia (120.9–133 mmol/L), hyperkalemia (6.4–9.1 mmol/L) and hyperbilirubinemia. His blood sugar, 17-hydroxyprogesterone, creatine kinase, very-long-chain fatty acid and gas chromatography mass spectrometry were normal. First hormone analysis showed adrenal insufficiency and elevated testosterone (Table 1). Ultrasound showed adrenal region got hypoechoic nodule that recognized as small adrenal gland. Genetic test found a pathogenic mutation of c.1169-1G>T in *NROB1* from his mother.

**Table 1:** Hormones levels and medication dosages

Age	Weight (kg)	ACTH (pg/mL)	Cortisol (nmol/L)	Aldosterone (pg/mL)	Renin (pg/mL)	LH (mIU/mL)	FSH (mIU/mL)	Testosterone (nmol/L)	Androstendione (ng/mL)	Hydrocortisone (mg/d)	Fludrocortisone	Episodes
1 d	3											
14 d	2.78							1.24				
22 d		172	5.94	243				3.85		3	0.05 mg bid	
47 d		11.7						5.72				
57 d		119						6.28				
68 d	4.6	320.3	179.68			1.32	6.96	5.93		12.5–20		Lower respiratory
77 d	5	3.29	> 1750	38.76		1.69	6.57	5.41		15		infection
3 mon			1613					2.72		8		
4.5 mon		3.71	1220					0.98				
6 mon	7.7	4.99	576					<0 .09		5	0.05 mg bid	
9 mon		34.78	497.6					< 0.09		5		
1 y 2 mon	11.2	176.5	627.6					< 0.09		5	0.05 mg bid	
1 y 7 mon		764	453					< 0.09				
2 y 1 mon		845.8	146.5					< 0.09				
3 y		169.7	531.1					< 0.09				
3 y 3 mon	20	443.7	159.9					<0 .087		6.25–10	0.05-0.025 mg bid	
4 y		127.3	480.2	62.01	88.3			< 0.088		10	0.033 mg bid	
4 y 5 mon	23	196.1	1.12	31.29	32.05			< 0.09		12.5	0.033 mg bid	
5 y 1 mon	29.2	> 2000	0.71	68.44	120.98	< 0.1	0.9	< 0.087	< 0.3	12.5	0.075 mg qd	

Reference value: adrenocorticotropic hormone (ACTH): 7.2–63.3 pg/mL, cortisol: 171–536 nmol/L, aldosterone: 40–310 pg/mL, renin: 4–38 pg/mL, luteinizing hormone (LH): 0.2–1.4 mIU/mL, follicle-stimulating hormone (FSH): 0.2–3.8 mIU/mL

After treatment with hydrocortisone, fludrocortisone and sodium salt replacement, his electrolyte gradually adjusted to normal in a month, and testosterone reduced to normal in 4 months (Table 1). Growth and development were under our observation as well. Delayed bone age and smaller testes were consistent with his disease.

Primary adrenal insufficiency combined with high testosterone tends to be diagnosed as congenital adrenal hyperplasia. From this case, we summarize DAX1 deficiency’s differential points as normal 17-hydroxyprogesterone, delayed bone age and confirmation of mutation in *NROB1*.Adrenal ultrasound also has certain value for distinguishing it from congenital adrenal hyperplasia.

Although most patients manifest sexual development failure, many reports broaden its clinical spectrum. Spontaneous or even precocious puberty happen but basically suspend in Tanner stage 2 to 3 [2, 3]. In this case, we observed elevated sexual hormones initially. Minipuberty was found in one case as well caused by a mutation of c.518del23 [4]. Another case was found with elevated testosterone since 9 months old with Trp291Arg in *NROB1* [5]. As testicular histological examination has been reported as intact in affected neonate [6], and testosterone can also be stimulated by human chorionic gonadotropin during early time [2], testicular structure and function can be normal but progressively impaired as increasing age. Excluding ectopic secretory tissue, testes can be reasonable origin for testosterone in early life.

Overall, although patients with DAX1 deficiency commonly present hypogonadism, individual case may have temporal elevated testosterone which confuses its diagnosis.
